# Doctors' Wills

**Published:** 1914-06-20

**Authors:** 


### Doctor's Wills.

Dr. Alexander James Main, M.D., of Alnwick, Northumberland, consulting physician to the Alnwick Infirmary, has left estate of the gross value of £38,014.

Dr. David Henry Small, aged eighty-nine, Upper Norwood, formerly a surgeon in the East India Company's Service, has left estate valued at £15,272.

